# Notch1 and Notch4 core binding domain peptibodies exhibit distinct ligand-binding and anti-angiogenic properties

**DOI:** 10.1007/s10456-022-09861-6

**Published:** 2022-11-15

**Authors:** Timothy Sargis, Seock-Won Youn, Krishna Thakkar, L. A. Naiche, Na Yoon Paik, Kostandin V. Pajcini, Jan K. Kitajewski

**Affiliations:** 1 Department of Physiology and Biophysics, University of Illinois College of Medicine, Chicago, IL 60612 USA; 2 Department of Pharmacology and Regenerative Medicine, University of Illinois College of Medicine, Chicago, IL 60612 USA; 3 University of Illinois Cancer Center, University of Illinois Chicago, Chicago, IL 60612 USA

**Keywords:** Notch, Angiogenesis, Inhibitor, Peptibody, Notch core binding domain

## Abstract

The Notch signaling pathway is an important therapeutic target for the treatment of inflammatory diseases and cancer. We previously created ligand-specific inhibitors of Notch signaling comprised of Fc fusions to specific EGF-like repeats of the Notch1 extracellular domain, called Notch decoys, which bound ligands, blocked Notch signaling, and showed anti-tumor activity with low toxicity. However, the study of their function depended on virally mediated expression, which precluded dosage control and limited clinical applicability. We have refined the decoy design to create peptibody-based Notch inhibitors comprising the core binding domains, EGF-like repeats 10–14, of either Notch1 or Notch4. These Notch peptibodies showed high secretion properties and production yields that were improved by nearly 100-fold compared to previous Notch decoys. Using surface plasmon resonance spectroscopy coupled with co-immunoprecipitation assays, we observed that Notch1 and Notch4 peptibodies demonstrate strong but distinct binding properties to Notch ligands DLL4 and JAG1. Both Notch1 and Notch4 peptibodies interfere with Notch signaling in endothelial cells and reduce expression of canonical Notch targets after treatment. While prior DLL4 inhibitors cause hyper-sprouting, the Notch1 peptibody reduced angiogenesis in a 3-dimensional in vitro sprouting assay. Administration of Notch1 peptibodies to neonate mice resulted in reduced radial outgrowth of retinal vasculature, confirming anti-angiogenic properties. We conclude that purified Notch peptibodies comprising EGF-like repeats 10–14 bind to both DLL4 and JAG1 ligands and exhibit anti-angiogenic properties. Based on their secretion profile, unique Notch inhibitory activities, and anti-angiogenic properties, Notch peptibodies present new opportunities for therapeutic Notch inhibition.

**Supplementary Information:**

The online version contains supplementary material available at 10.1007/s10456-022-09861-6.

## Introduction

Angiogenesis is a tightly regulated multi-step process that defines the development of new blood vessels emanating from existing vessels. Under physiological conditions, this action is confined to embryonic and postnatal development as well as tissue growth and wound healing during the adult life. Angiogenic aberrations have been implicated in several pathologies such as in cancer, vascular malformations, and retinopathy. Understanding how angiogenesis contributes to tumor growth and inflammation has long been a key area of interest for therapeutic intervention [1]. In the absence of vascular support, tumors rarely develop past 2mm^3^, demonstrating the critical role angiogenesis plays in the development of tumor growth [2]. This vulnerability in turn pressures tumors to up-regulate pro-angiogenic factors and recruit nearby endothelial cells to maintain growth, and ultimately, metastatic spread.

Notch signaling functions in angiogenesis through the regulation of endothelial cell-fate decisions, often control separate angiogenic pathways, such as vascular endothelial growth factor receptor (VEGFR) signaling, to carry out this function [3, 4]. Mammals express four homologous notch receptors (Notch 1–4) and five ligands [Jagged (JAG) 1, 2 and delta-like ligand (DLL) 1,3, 4]. Ligand–receptor interactions cause subsequent cleavage of the Notch receptor and translocation of the Notch intracellular domain (NICD) to the nucleus, leading to transcription of downstream target genes. It has been well documented that the Notch ligand DLL4 acts through endothelial Notch as a negative regulator of VEGF receptors to restrict angiogenesis, thus producing appropriate number of functional vessels [3–5]. The Notch ligand, JAG1, plays a pro-angiogenic role but the mechanisms of JAG1-Notch signaling in endothelial cells are not well understood [6, 7]. Tumor vasculature regularly exploits angiogenic pathways that respond to hypoxia-regulated VEGF, which in turn up-regulates DLL4 that then activates Notch signaling. Thus, the involvement of Notch signaling in pathological angiogenesis intersects with VEGFR signaling and has marked it as a potential target for controlling this process.

At the time of writing, there is no clinically approved Notch targeted therapeutic for use in oncology. Previous approaches to globally inhibit the activation of the Notch pathway have raised safety concerns due to toxicity. The most prominent class of Notch inhibitors are those that target γ-secretase. γ-secretase inhibitors (GSI) block the cleavage of Notch and the subsequent translocation of the intracellular domain of Notch (ICN) to the nucleus, inhibiting Notch signaling. Aberrant activation of Notch1 in T cell acute lymphoblastic leukemia (T-ALL) patients led to the deployment of GSIs for use in clinical trials [8–11]. However, most patients suffered from gastrointestinal symptoms such as diarrhea in a dose-dependent manner, making treatment sub-optimal [12]. Animal studies have further confirmed that systemic inhibition of Notch signaling results in gastrointestinal toxicity due to accumulation of secretory goblet cells in the intestine [13–15]. The development of DLL4 neutralizing antibodies was a promising next step in targeting the endothelial Notch1 signaling axis without the toxicity issues associated with global Notch blockade. The role of DLL4/Notch1 signaling in the development of the tumor vasculature has been studied extensively where it has been shown that Tip Cell formation, the initial cellular step of angiogenesis, is inhibited by DLL4–Notch signaling. Thus, the rationale to inhibit DLL4–Notch1 signaling appears paradoxical as its inhibition decreases tumor growth by triggering excessive but poorly perfused tumor vessels. Despite its promise, anti-DLL4 therapy evaluated in pre-clinical animal models resulted in pathological changes in the liver as well as severe vascular neoplasms [16].

Development of new and safe approaches for targeting the Notch pathway remains a critical clinical problem, based on the potential to inhibit several types of malignancies by restricting functional vessels. Our previous work with Notch decoys demonstrated effective inhibition of tumor growth with minimal gastrointestinal toxicities associated with Notch inhibition [17]. These decoys comprised a varying number of EGF-like repeats of the Notch1 extracellular domain fused to IgG Fc and function as inhibiting peptibodies. This work, however, was performed using adenoviral administration of decoy-producing vectors to produce and evaluate the activity of Notch decoys in mice. While adenoviral vectors have been approved for some human trials, purified proteins provide control over dosage and carries less risk of inappropriate immune response than with viral administration. Here, we explore a peptibody-based approach, using biologically active peptides constituting select Notch EGF-like repeats fused with IgG Fc. This strategy presents a novel alternative to therapeutic antibodies while preserving certain antibody-like characteristics, such as increased binding affinity and increased plasma stability arising from the dimerization of Fc fragments [18, 19]. Peptibodies, like traditional antibodies, can efficiently interact with Fc receptors to induce an innate immune response from natural killer cells and macrophages, creating a synergistic therapeutic effect in some contexts [20].

The Notch peptibodies, or Notch decoys, we have developed comprise five EGF-like repeats spanning the known core binding domain of the Notch1 receptor and the predicted corresponding core binding domain of the Notch4 receptor. Here, we show that these Notch decoys can be purified as active proteins that bind to Notch ligands with high affinity. The Notch1 decoy demonstrated inhibition of Notch target gene expression and anti-angiogenic properties in vitro and in developmental mouse models, suggesting that it may represent a therapeutic option for targeting Notch signaling.

## Results

### Design and characterization of Notch peptibodies

**Fig. 1:**
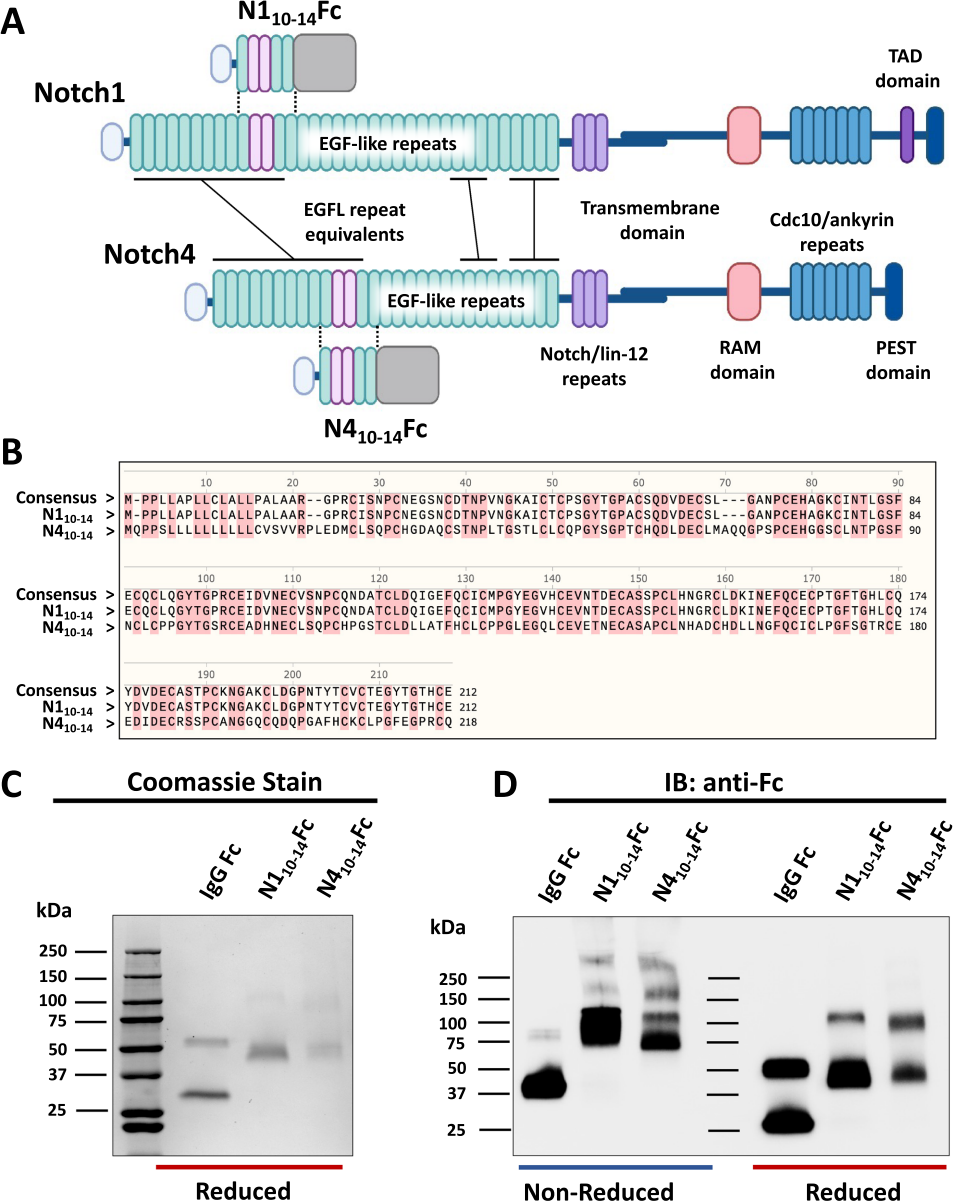
Construction and expression of Notch decoys. **A** Notch decoys are composed of specific EGF-like repeats (10–14) of the Notch1 or Notch4 extracellular domain fused to the human Fc domain. **B** Amino acid alignment of EGFs 10–14 of the Notch1 and Notch4 receptor, respectively. **C** Coomassie stained TGX-Gel analysis. Purified proteins were loaded under reducing conditions. **D** Western blot analysis. 100 ng of purified protein were resolved on TGX gels under non-reducing or reducing conditions and immunoblotted with Fc-specific antibody

The extracellular domain of the four Notch proteins is composed of a varying number of EGF-like repeats. For Notch1, structure–function analysis coupled with high-resolution crystal structures have identified EGF-like repeats 11–12 as critical for receptor–ligand interaction [21–24]. In contrast to the Notch1 receptor, the core ligand-binding domain of Notch4, an endothelial-specific Notch gene [25], has not been fully characterized. While Notch4 is the most divergent of the four mammalian Notch receptors, EGFs 11–12 are highly homologous between Notch1 and Notch4. Of the four human Notch proteins, Notch1, Notch2, and Notch4 have the highest levels of conservation between EGF-like repeats 10–14, while a notable divergence appears in Notch3, where EGFs 7–10 of the Notch3 receptor best corresponds to EGFs 11–13 of Notch1 (Fig. S1A) [26]. Further, protein alignment of the human and murine Notch receptors across EGFs 10–14 demonstrates high identity and similarity among these two mammalian species (Fig. S1B). Here, we generated recombinant fragments of the human Notch1 and Notch4 extracellular domains containing the coding sequences of Notch1 and Notch4 EGF-like repeats 10–14 fused to human IgG Fc, referred as N1_10-14_Fc and N4_10-14_Fc in our data. The sequence alignment of these two Notch decoys demonstrated regions of high conservation across EGFs 10–14, including EGFs 11 and 12 (Fig. 1A, B).

N1_10-14_Fc and N4_10-14_Fc proteins were produced in HEK293F cells upon transfection with the corresponding expression construct. The secreted proteins were subsequently column purified and resolved in 4–20% SDS-PAGE gels stained with Coomassie, showing approximate molecular weights (MW) of N1_10-14_Fc and N4_10-14_Fc with an expected band at ~ 50 kDa in reducing conditions (Fig. 1C). To confirm the identity of the proteins, western blotting was performed using antibodies specific for human IgG Fc in both non-reducing and reducing conditions, showing specific bands at ~ 100 kDa in non-reducing and ~ 50 kDa in reducing conditions (Fig. 1D). No evidence of cleavage between the Notch EGF-like repeats and Fc domain or other forms of degradation were apparent. As expected, detection of native protein under non-reducing conditions was at double the predicted molecular weight, indicating dimerization of the IgG Fc. To accurately assess their oligomeric states, mass photometry was utilized to evaluate N1_10-14_Fc and N4_10-14_Fc proteins. Both Notch decoys displayed an oligomeric mixture dominated by dimers at 100 kDa (Fig. S2).

### Both N1_10–14_Fc and N4_10–14_Fc bind to DLL4 and JAG1

**Fig. 2:**
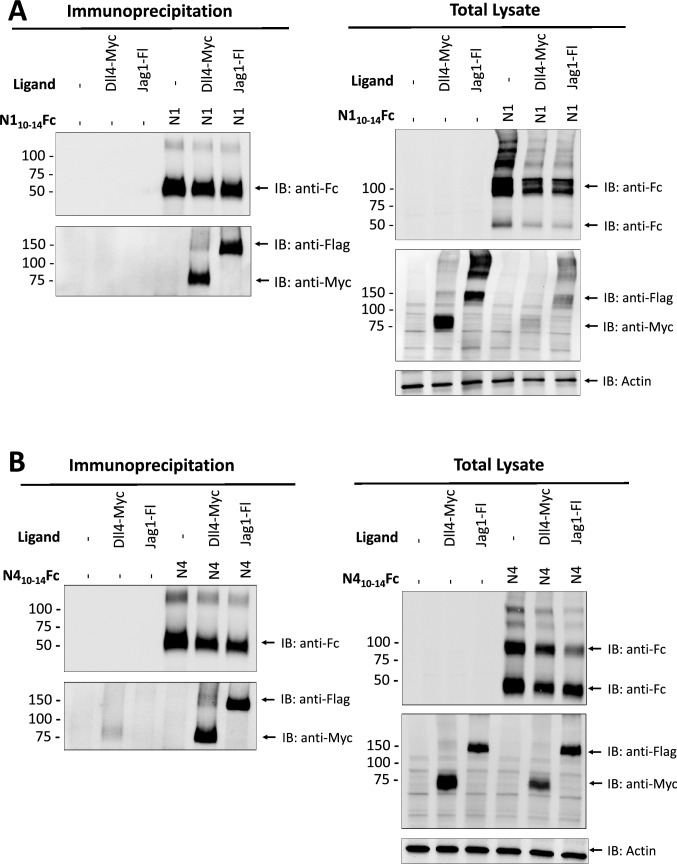
Binding of Notch ligands and Notch decoys examined by co-immunoprecipitation. Co-immunoprecipitation assays show interaction of Notch ligands DLL4 and JAG1 with Notch peptibodies. **A** N1_10-14_Fc and full-length DLL4-MYC or JAG1-FLAG were transiently co-transfected into HEK-293Ts. Protein A/G beads were used to immunoprecipitate N1_10-14_Fc acting as the “bait” protein from whole cell lysates. Binding of N1_10-14_Fc and Notch ligands were determined by immunoblot using anti-Fc, anti-FLAG, and anti-MYC antibodies. **B** N4_10-14_Fc was evaluated similarly to panel A

**Fig. 3:**
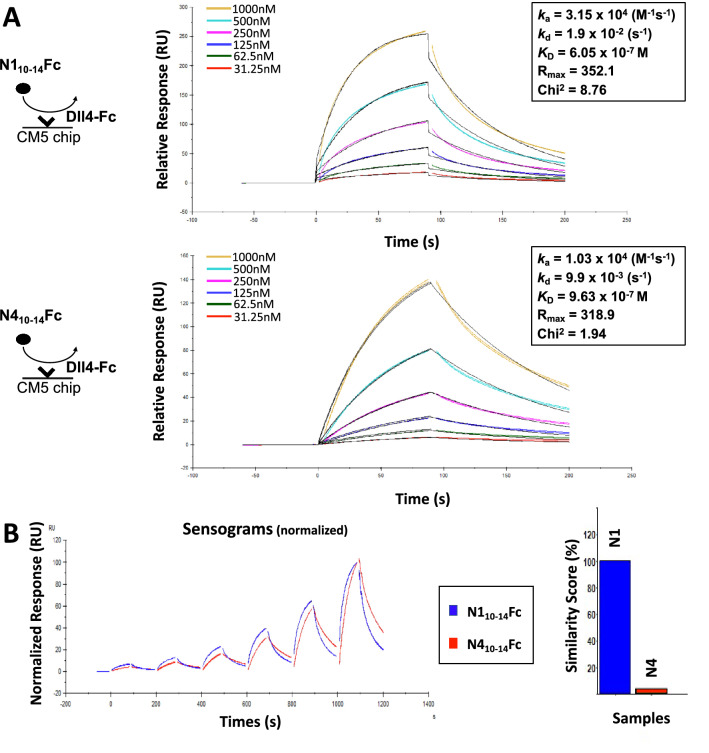
Binding of Notch ligands and Notch decoys examined by SPR. SPR measurements show binding affinity of N1_10-14_Fc and N4_10-14_Fc to DLL4. **A** Recombinant Fc-tagged hDLL4 was immobilized on the sensor chip using amine coupling and multi-cycle kinetic experiments were performed using increasing concentrations of either N1_10-14_Fc or N4_10-14_Fc. **B** Normalization of the N4_10-14_Fc sensorgram to N1_10-14_Fc. The resulting sensorgrams were normalized using Biacore sensorgram fitting algorithms and similarity scores

We asked if the homologous ligand-binding domain of Notch4 is sufficient to bind to Notch ligands DLL4 and JAG1. It has been well documented that for Notch1, EGF-like repeats 11 and 12 correspond to the core binding domain and are alone sufficient for ligand interaction [21–24]. To date, there has been little evidence that Notch4 binds to ligands DLL4 and JAG1. To confirm binding specificity, N1_10-14_Fc, N4_10-14_Fc, full-length FLAG-tagged JAG1, and full-length Myc-tagged DLL4 were co-expressed in 293 T cells and co-immunoprecipitation was performed with Notch decoys acting as the “bait” protein. After pulldown, N1_10-14_Fc and N4_10-14_Fc co-immunoprecipitated with both DLL4 and JAG1, validating pan-ligand binding (Fig. 2A, B). We utilized surface plasmon resonance (SPR) spectroscopy to characterize and quantify the interactions between these proteins. Recombinant Fc-tagged hDLL4 and hJAG1 were immobilized on the sensor chip using amine coupling and multi-cycle kinetic experiments were performed using increasing concentrations of either N1_10-14_Fc or N4_10-14_Fc. As a control, recombinant IgG Fc was immobilized on the sensor chip. Recombinant hDLL4 interacted with N1_10-14_Fc and N4_10-14_Fc with a dissociation constant (*K*_D_) of 6.05 × 10^–7^ M and 9.625 × 10^–7^ M, respectively (Fig. 3A). Based on previous literature, we had predicted N1_10–14_Fc would bind to DLL4 and JAG1. We report here for the first time a conserved binding domain within Notch4 that promotes interaction with DLL4 and JAG1. A notable feature of the sensorgrams between the two decoys shows N1_10–14_Fc binding to hDLL4 at a fast association and disassociation rate compared to N4_10-14_Fc (Fig. 3B), indicating different binding mechanics. Although no interaction was measured between hJAG1 and N1_10-14_Fc or N4_10-14_Fc (Fig. S3) in SPR-based binding assays, this result aligns with prior published reports [22]. It has been theorized that the interaction of the Notch1 extracellular domain and that of JAG1 is weak and requires a pulling force to stabilize JAG1 into confirmations needed for interaction. We conclude that Notch decoys can readily bind to members of Delta-like or Jagged/Serrate-class Notch ligands.

### N1_10–14_Fc suppresses endothelial Notch signaling

**Fig. 4:**
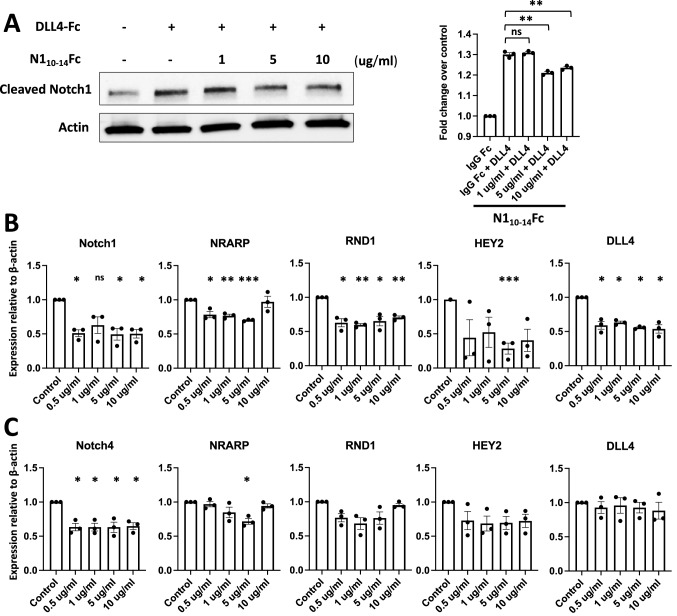
N1_10–14_Fc suppresses endothelial Notch signaling. **A** N1_10-14_Fc inhibits DLL4-induced cleavage of the Notch1 receptor. HUVECs were plated onto 1 μg/mL of recombinant hDLL4 in the presence of N1_10-14_Fc or IgG Fc isotype control for 24 h and quantitated for cleaved Notch1 by Western blot. (B, C) RT-qPCR analysis of Notch decoy-induced gene changes in HUVECs. Cells were treated with either Notch peptibody or IgG Fc at indicated concentrations for 24 h. **B** Expression of targets of canonical Notch signaling in HUVECs treated with N1_10-14_Fc. **C** Expression of targets of canonical Notch signaling in HUVECs treated with N4_10-14_Fc. For all figures, error bars represent standard error of mean (SEM) and ***P value < 0.001, **P value < 0.01, *P value < 0.05

Interaction of endothelial Notch with Notch ligands, such as DLL4, causes the cleavage of the intracellular domain of Notch1 and subsequent translocation to the nucleus. Notch decoys that inhibit DLL4–Notch interactions would be expected to inhibit cleavage of endogenous Notch1 expressed on the surface of endothelial cells. To evaluate whether Notch decoys can block DLL4-mediated Notch1 activation, we seeded HUVECs onto DLL4-coated plates and subsequently dosed with increasing concentrations of N1_10-14_Fc. At the highest doses tested, N1_10-14_Fc reduced the DLL4-induced cleavage of endogenous Notch1 expressed in HUVECs (Fig. 4A).

Once the NICD enters the nucleus, it interacts with the transcription factor RBPJ/CSL to regulate expression of canonical Notch target proteins. To directly test whether Notch decoys affect the canonical Notch signaling pathway, a crucial regulator in endothelial cells, we examined the inhibitory effects of N1_10-14_Fc and N4_10-14_Fc on the Notch pathway in HUVECs. Increasing concentrations (0, 0.5, 1, 5, and 10 ug/ml) of either IgG Fc, N1_10-14_Fc, or N4_10-14_Fc were used to treat HUVECs for 24 h. Differences in mRNA expression of Notch target genes were then examined by RT-qPCR. Compared to the control group, N1_10-14_Fc significantly downregulated Notch target genes NRARP, HEY2, RND1, DLL4, and Notch1 at multiple concentrations (Fig. 4B). N4_10-14_Fc decoy downregulated Notch target genes to a lesser degree in HUVECs (Fig. 4C).

### N1_10–14_Fc inhibits angiogenesis in vitro

**Fig. 5:**
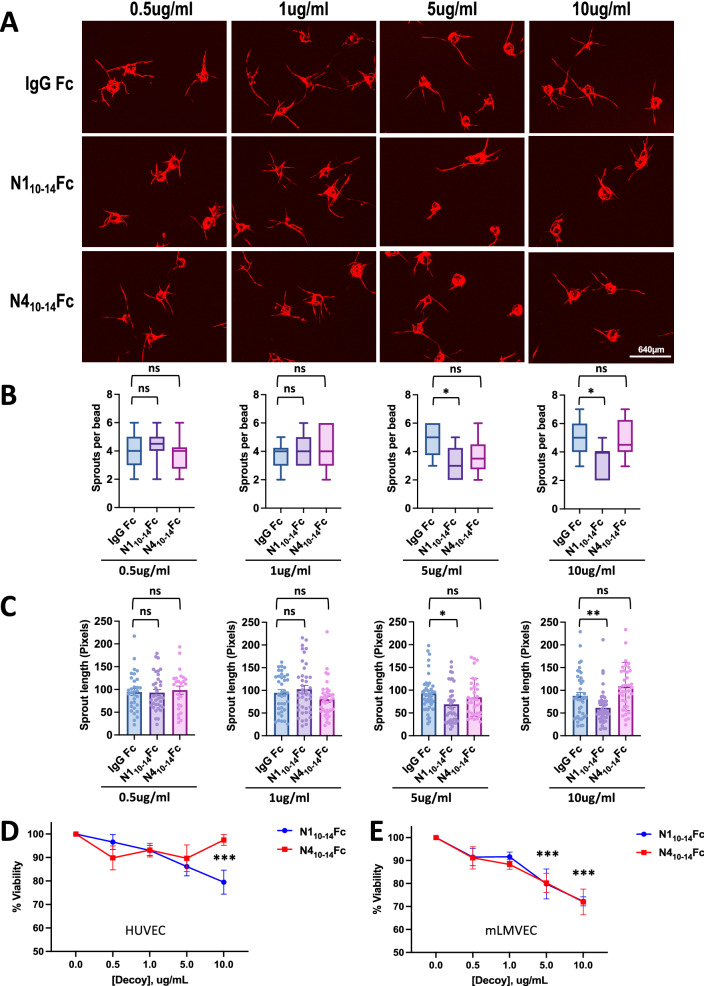
N1_10–14_Fc affects endothelial viability and modulates angiogenesis. **A** Representative images of Fibrin bead assays (FiBA). HUVEC-coated beads were embedded in fibrin gel with increasing doses of human IgG Fc, N1_10-14_Fc, or N4_10-14_Fc for 12 days. After 12 days, N1_10-14_Fc significantly reduced both sprout number and sprout length at dosages of 5 and 10ug/ml. No significant effect was seen with N4_10-14_Fc at any dosage. **B** Quantification of mean sprout number per bead for treated HUVECs. Box-and-whisker plots show median, minimum, and maximum values. **C** Quantification of mean sprout length for treated HUVECs. **D** Cell viability assay of HUVECs treated with increasing dose of IgG Fc, N1_10-14_Fc, or N4_10-14_Fc for 72 h. IgG Fc-treated control group was set at 100% and was compared with that of peptibody-treated groups. **E** Identical experiments conducted with mLMVEC. **P value < 0.01, *P value < 0.05

Angiogenesis is a tightly controlled, multi-step process in endothelial cells that involves proliferation, cell migration, and tube formation. To assess the effects of our Notch decoys on endothelial sprout formation, a three-dimensional in vitro assay was used. Cytodex beads were coated with HUVECs and subsequently embedded into a fibrinogen matrix. To support HUVEC growth, fibroblast cells were cultured on top of the matrix to provide growth factors. In this assay, endothelial sprouts grow out from the bead, mimicking the nascent stages of angiogenesis. We evaluated the number of outgrowths and their corresponding length in HUVECs treated with increasing concentrations of either IgG Fc, N1_10-14_Fc, or N4_10-14_Fc. After treatment with N1_10-14_Fc, the number of angiogenic sprouts and the length of the newly formed sprouts were reduced at concentrations of 5 and 10 ug/ml of the Notch decoy (Fig. 5A–C). No significant reduction was seen in either sprout length or sprout number after treatment with N4_10-14_Fc (Fig. 5A–C).

To assess if Notch decoys have cytotoxic effects on human or mouse endothelial cells, we treated cells with increasing concentrations of either IgG Fc, N1_10-14_Fc, or N4_10-14_Fc for 72 h. The viability of HUVECs was dose-dependently inhibited by N1_10-14_Fc but not by N4_10-14_Fc (Fig. 5D). The viability of mouse lung microvascular endothelial cells (mLMVEC) was dose-dependently inhibited by both peptibodies. We asked if the anti-angiogenic effect observed in vitro is due in part to a migration defect or solely viability. Previous literature has demonstrated that inhibition of DLL4–Notch signaling has been shown to induce the migration of endothelial cells. To understand the role of Notch decoys on endothelial cell migration, we utilized a scratch wound-healing assay in which the extent of migration of cells into the scratched areas was examined. HUVECs or mLMVECs treated with either IgG Fc, N1_10-14_Fc, or N4_10-14_Fc were assessed and no migration alterations were detected (Fig. S4). These results indicate that N1_10-14_Fc, but not N4_10-14_Fc, modulates human endothelial viability and both peptibodies reduce mouse endothelial viability.

To assess whether Notch peptibodies affect other cell types, we examined T cell acute lymphoblastic leukemia (T-ALL) cells which require Notch signaling for survival [8–11]. N1_10-14_Fc and N4_10-14_Fc showed no significant effects on survival of human T-ALL KopTK cells or mouse T6E cells (Fig. S5).

### N1_10-14_Fc inhibits murine retinal angiogenesis

**Fig. 6:**
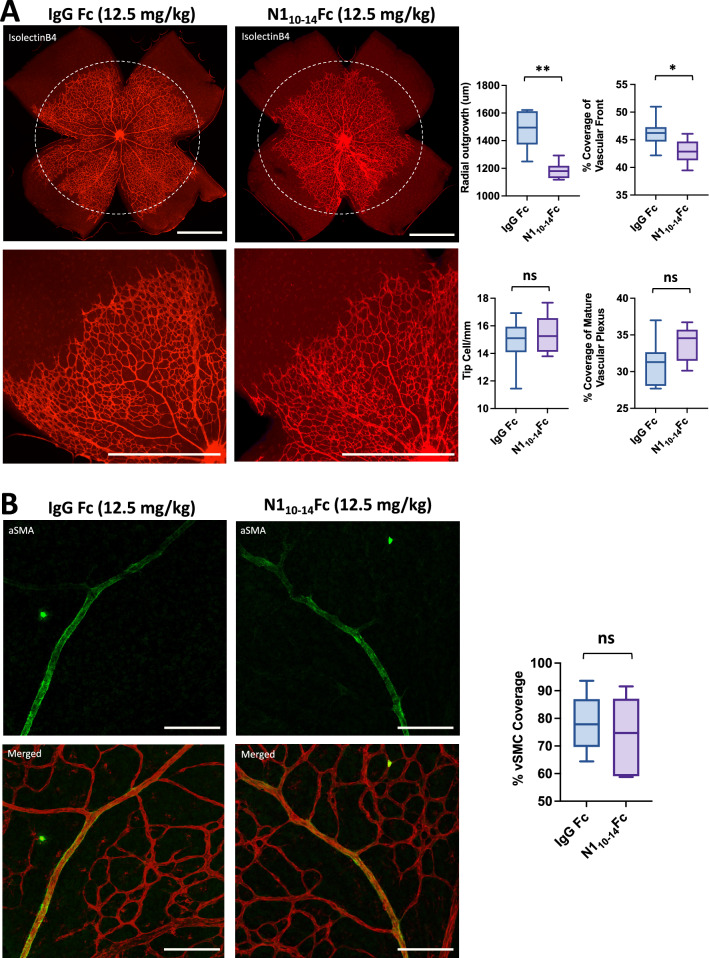
N1_10–14_Fc inhibits retinal angiogenesis in murine neonates. C57BL/6 mice were injected intragastrically with 12.5 mg/kg of recombinant N1_10-14_Fc peptibody or IgG Fc for three days postnatally (P1-P3). **A** Representative images and quantification of postnatal day 5 (P5) retinal vasculature stained with Isolectin B4 (red). Radial outgrowth and percent vascular coverage near the angiogenic front were reduced in N1_10–14_Fc-treated mice (*N* = 7–8), while tip cell density and percent vascular coverage of the mature plexus were not statistically altered. Scale bars: 1000 μm. **B** Representative images and quantification of postnatal day 5 (P5) retinal vasculature stained with Isolectin B4 (endothelium, red) and α-SMA (vascular smooth muscle cells, green). No difference was observed in the percentage of smooth muscle coverage in control and N1_10-14_Fc-treated mice (*N* = 4). Scale bars: 106 μm. Box-and-whisker plots show median, minimum, and maximum values. **P value < 0.01, *P value < 0.05. (Color figure online)

We assessed N1_10-14_Fc treatment during postnatal murine angiogenesis to better understand how peptibody-based Notch inhibitors effect angiogenesis in vivo. To deliver the recombinant proteins, either human IgG Fc or N1_10-14_Fc were injected intragastrically into murine neonates. Compared to the Fc-treated group, N1_10-14_Fc showed a reduction in both the vascular area of the angiogenic front and radial vascular outgrowth (Fig. 6A). Further, filopodia-extending endothelial sprouts, termed Tip Cells, were observed to be no more abundant in mice treated with N1_10-14_Fc than Fc. While not the focus of this investigation, we observed that approximately half the mice treated with N1_10-14_Fc exhibited unusual enlargement of retinal veins (Fig. S6), which warrants future investigation.

In some vascular development settings, Notch ligands JAG1 and DLL4 play a crucial role in the recruitment of vascular smooth muscle cells to nascent arteries during the maturation process of angiogenesis [5, 17, 27–29]. Vascular smooth muscle cell coverage of mice treated with N1_10-14_Fc remained unchanged compared to the control group (Fig. 6B), indicating that N1_10-14_Fc inhibited angiogenesis with no effect on vascular remodeling at this time point. These results suggest that N1_10-14_Fc can cause inhibition of angiogenic vessels in vivo.

## Discussion

To date, there are no approved Notch inhibitors for use in oncology after decades of development of numerous small molecules and antibody-based therapeutics designed to target Notch signaling [30]. The absence of approved Notch inhibitors is a barrier to therapeutic manipulation of the critical role of the Notch pathway in tumor angiogenesis and immunology. Thus, development of new approaches to safely target the Notch signaling pathway remains a critical clinical problem that is currently unaddressed.

In order to combine the superior pharmacokinetics of antibodies to the targeting capabilities of peptides, fusions between the two have been previously developed [17]. These fusions, termed peptibodies, are comprised of IgG Fc and peptides with binding properties to the target protein. This strategy presents a novel alternative to therapeutic antibodies while preserving antibody-like characteristics, such as increased binding affinity and increased plasma stability arising from the dimerization of Fc fragments [18, 19]. In this report, we investigated a novel alternative means to targeting the Notch signaling pathway using a peptibody-based approach by combining the Notch core binding domain with the Fc domain of human IgG. The peptides described here comprise the human Notch1 and Notch4 extracellular domains containing the coding sequences of Notch1 and Notch4 EGF-like repeats 10–14, respectively. This region has been identified as critical for receptor–ligand interaction in Notch1. While no binding domain had been previously identified in Notch4, EGF-like repeats 10–14 are highly homologous between Notch1 and Notch4, and thus we reasoned a similar binding domain may be present. We chose to construct Notch peptibodies comprising the receptors of Notch1 and Notch4 due to loss and gain-of-function evidence that implicates these two endothelial Notch proteins in the regulation of angiogenesis in the vasculature [29, 31–34].

Biophysical studies on the extracellular domain of Notch proteins have been limited because of the size and low expression levels of the proteins, impeding full characterization of receptor–ligand binding [35]. Due to the significantly improved secretion properties of the Notch peptibodies, we were able to characterize the ligand-binding properties of N1_10-14_Fc and N4_10-14_Fc to Notch ligands DLL4 and JAG1 (Fig. 2, 3). Using surface plasmon resonance spectroscopy coupled with co-immunoprecipitation assays, we show that both Notch1 and Notch4 peptibodies have pan-ligand-binding capabilities (DLL4, JAG1) with favorable binding properties. Further, we demonstrate for the first time utilizing our N4_10-14_Fc peptibody that Notch4 maintains a conserved binding domain within this region. Nonetheless, N1_10-14_Fc and N4_10-14_Fc showed highly dissimilar binding kinetics to DLL4, demonstrating that these homologous regions interact differently with ligands. Several EGF-like repeats of the Notch ECDs contain glycosylation sites that play a crucial role in signaling by affecting folding of the Notch receptor that influences receptor–ligand interactions [35–38]. It has been shown that optimal ligand–receptor binding requires proper posttranslational modifications on specific EGF-like repeats, such as EGF 12 on the Notch1 receptor, with O-fucose [35, 39–41]. While posttranslational modifications of EGF-like repeats on the Notch1 receptor have been partially characterized, those on the Notch4 receptor remain unexamined. Disparities in glycosylation states of N1_10-14_Fc and N4_10-14_Fc could explain the observed differences in binding mechanics to DLL4 and warrant future studies.

The Notch peptibodies with core ligand-binding domains of Notch1 and Notch4 bound to Notch ligands with high affinity, thus we reasoned that N1_10–14_Fc would operate as decoys and compete with endogenous Notch proteins, subsequently reducing activation of downstream signaling. Here, we observed that HUVECs treated with N1_10–14_Fc blocked cleavage of the Notch1 intracellular domain when challenged with ligand DLL4. Further, treatment of N1_10–14_Fc was shown to significantly downregulate canonical Notch target genes in endothelial cells while treatment of N4_10–14_Fc demonstrated a more subdued effect on canonical Notch targets when evaluated. Treatment of either N1_10-14_Fc or N4_10-14_Fc showed target gene reduction through a dose-independent response (Fig. 4). However, we note that binding kinetics, cell viability, and endothelial sprouting assays all showed stronger responses only at the higher doses (Fig. 3 and Fig. 5). These results suggest that these Notch target genes are sensitive to even low doses of peptibody inhibition, highlighting how transcriptional responses may be the most sensitive to the addition of Notch peptibodies.

Due to the significant effect of N1_10-14_Fc on Notch-mediated gene expression, we examined whether Notch peptibodies affect cell viability in monolayer culture. We found that N1_10-14_Fc, but not N4_10-14_Fc, showed cytotoxic effects on monolayer HUVEC cells in a dose-dependent manner. When evaluated in a 3D sprout formation assay, N1_10-14_Fc, but not N4_10-14_Fc, showed a reduction in both neo-angiogenic sprout length and number, demonstrating anti-angiogenic properties. While N4_10-14_Fc bound to both DLL4 and JAG1, our results indicate that it does not have the same anti-angiogenic properties as that of N1_10-14_Fc. It has been demonstrated that targeted deletion of Notch1 in vivo yielded severe developmental defects, such as dysregulated vascular angiogenesis [29, 31–34]. In contrast, targeted deletion of the Notch4 gene in vivo generated an anti-angiogenic phenotype [25, 33, 34]. A double knockout study of both Notch1 and Notch4 revealed a more severe dysregulation of vascular angiogenesis than the Notch1 single knockout alone [33]. These genetic studies suggest that Notch4 may play a redundant role in developmental angiogenesis but also that Notch4 is moderately pro-angiogenic, consistent with what we observed with the treatment of N4_10-14_Fc peptibodies. Conversely, some studies suggest that Notch4 plays a specific role in specific endothelial pathologies, suggesting that treatment with N4_10-14_Fc peptibodies may show stronger effects under pathological conditions [32, 42–44].

Development of the neonatal mouse retina is a well-established animal model for angiogenesis, which has been applied in numerous studies when evaluating potential anti-angiogenic agents. In this study, we evaluated how purified Notch peptibodies affect angiogenesis when administered to mice*.* Marked reduction in both radial outgrowth and vascular density at the angiogenic front was observed in mice treated with N1_10-14_Fc, confirming our anti-angiogenic observations in vitro. The role of Notch ligands in the regulation of vessel maturation during vessel development has been established in some vascular models while less established in others. To better understand the role of Notch ligands in the developing retina, we evaluated the effects of vascular smooth muscle cell coverage in mice treated with N1_10-14_Fc. No change in vascular smooth muscle cell coverage was seen in mice treated with N1_10-14_Fc when compared to the control group, indicating that N1_10–14_Fc delayed angiogenesis while preserving recruitment and differentiation of vascular smooth muscle cells during vascular development. Interestingly, we noted enlargement of veins in half of mice treated, a unique phenotype that can be attributed to the inhibition of Notch signaling [3].

It is interesting to note that N1_10-14_Fc did not result in significant hyper-sprouting, commonly found when treating mice with GSIs or DLL4-specific inhibitors. Pharmacological inhibition of DLL4/Notch signaling has been shown to trigger excessive angiogenic sprouting and results in an abnormally dense and poorly perfused vascular plexus during retinal development [45]. In contrast, inhibition of JAG1/Notch has been shown to reduce angiogenesis and mural cell coverage in the developing retina [17]. Despite being an inhibitor to both DLL4 and JAG1, treatment of N1_10-14_Fc in neonatal retinal angiogenesis conferred a unique anti-angiogenic phenotype without increase in tip cell formation or reduction of vSMC coverage. Thus, Notch1 peptibodies presents us with an alternative class of inhibitor to the Notch signaling pathway that allows us to reduce angiogenesis while retaining vascular maturation and functionality.

Taken together, Notch peptibodies consisting of the core ligand-binding domain of Notch ECDs act as decoys by binding to Notch ligands, downregulate Notch signaling, and decrease angiogenesis, demonstrating the anti-angiogenic effects of these agents. Further studies are warranted to evaluate the potential of these purified Notch decoys in pathological angiogenesis, such as in inflammatory diseases and cancer, and to determine their toxicity profile. Considering the ability to purify these multi-ligand-binding Notch decoys, these agents should eventually be available for evaluation in clinical studies in the numerous settings where excess Notch signaling drives human disease.

## Methods

### Expression and purification of notch decoys

Expression vectors of N1_10-14_Fc and N4_10-14_Fc were transfected in HEK Expi293 cells using the Expi293 Expression System (Thermo Fisher Scientific). Notch decoys were subsequently purified from cultured media by HiTrap rProtein A FF (GE Healthcare) affinity chromatography. Eluted fractions were collected and immediately dialyzed to exchange buffer into PBS. Protein was concentrated in Vivaspin 20 10,000 MWCO concentrators (Sartorius).

### Cell lines

All cell cultures were maintained at 37 °C in a mixture of 5% CO2 and 95% humidified air. HUVECs isolated from human umbilical veins (Lonza) were grown in EGM-2 Media (Lonza) on culture plates coated with rat tail type I collagen (354,236; BD Biosciences, Franklin Lakes, NJ). HEK293T cells were purchased from ATCC and maintained DMEM (Gibco, Cat No. 11–995-073) with 10% FBS. Normal human lung fibroblasts (NHLFB) were purchased from Lonza and cultured with fibroblast growth media (Lonza).

### Western blots

Cells were lysed in ice cold cell RIPA buffer (9806; Cell Signaling) containing 1 × protease inhibitor (Thermo Fisher Scientific, 78,430), 1 × phosphatase inhibitor (Thermo Fisher Scientific, 78,420), and 1 mM of DDT, and western blots were performed. Primary antibodies against cleaved Notch1 (Val1744), FLAG (D6W5B**)**, MYC, and Actin (13E5**)** were from Cell Signaling Technology (Danvers, MA) and incubated in blocking buffer (5% BSA, and 1 × TBST 0.1% Tween 20). Gel images were obtained using the Chemidoc MP imaging system (Bio-Rad), and quantitation was performed using ImageJ.

### Co-immunoprecipitation assay

N1_10-14_Fc or N4_10-14_Fc and full-length DLL4-MYC or JAG1-FLAG were transiently co-transfected into HEK-293 T cells using Lipofectamine 2000. A crosslinking agent, Disuccinimidyl glutarate (20,593; Thermo Fisher Scientific), was added to the culture 24 h after transfection at a final concentration of 20 nmol/ml and incubated for 30 min. The cells were subsequently lysed in 100 μl of 1 × cell lysis buffer from Cell Signaling (#9803). The lysate was pulled down by 20 μl of Protein A/G magnetic beads (Thermo Fisher Scientific). To reverse the crosslink prior to western blot analysis, the immunocomplex was treated with 50 μmol/ml dithiothreitol (DTT) and boiled for 4 min before electrophoresis.

### Affinity analysis

The binding kinetics of N1_10-14_Fc and N4_10-14_Fc were analyzed using a Surface Plasmon Resonance (SPR)-based assay on the Biacore T200 system (GE Healthcare). Human IgG Fc (Sino Biologics) was firstly immobilized onto a CM5 biosensor chip. Then, an appropriate concentration of hDLL4-Fc (Sino Biologics) and hJAG1-Fc (Sino Biologics) was captured to the surface at a Response Unit (RU) of up to 20,000. Finally, various concentrations of N1_10-14_Fc and N4_10-14_Fc were passed through the chip with running buffer [1 × HBS-N (10 mM HEPES, 150 mM NaCl) with 0.005%Tween 20,1 mM CaCl2, 2 mM MgCl2, pH7.4]. After each reaction, the captured ligands and analyte were removed by regeneration buffer [1 × HBS-N (10 mM HEPES, 150 mM NaCl) with 0.005%Tween 20, 1 mM CaCl2, 2 mM MgCl2, pH7.4]. The whole reaction was conducted at 25 °C and flow rate of 25 μl/min. Sensorgrams of each concentration were obtained and analyzed by Biacore evaluation software (GE Healthcare). The equilibrium constant *K*_*D*_ was calculated from the ratio of dissociation rate constant *k*_*d*_ to association rate constant *k*_*a*_ (*k*_*d*_/*k*_*a*_).

### Quantitative real-time polymerase chain reaction (qRT-PCR)

Total RNA from HUVECs treated with either human IgG Fc (Sino Biologics), N1_10-14_Fc or N4_10-14_Fc was collected after 24 h as recommended by the manufacturer using Qiagen RNEASY. Complementary DNA (cDNA) synthesis was performed using approximately 1 μg RNA per 20 μl using a cDNA reverse transcription kit (Thermo Fisher Scientific). Real-time PCR was performed on an ViiA 7 real-time PCR system (Life Technologies) using SYBR Green.

### Cell viability assay

For endothelial cells, 96-well plates were seeded with 4 × 10^3^ HUVEC or mLMVEC cells (Lonza), with indicated concentrations of either human IgG Fc (Sino Biologics), N1_10-14_Fc, or N4_10-14_Fc. Each concentration is represented by six replicates. For T-ALL cells, KopTK or T6E T-ALL cells were seeded at 8 × 10^3^ cells per well in a 96-well plate in RPMI media and treated with the indicated concentration of IgG Fc, N1_10–14_Fc, or N4_10–14_Fc. After incubation for 72 h, cell viability was determined by XTT assay (Biotium).

### Scratch wound-healing assay

HUVECs (Lonza) treated with cell-tracker CMFDA dye (Thermo Fisher) were seeded in 24-well plates coated with rat tail type I collagen (354,236; BD Biosciences, Franklin Lakes, NJ) and “scratch-wounded” using a 200 μl pipette tip. After wounding, cells were treated with different concentrations (5, 10 ug/ul) of either IgG Fc, N1_10–14_Fc, or N4_10–14_Fc. After approximately 14 h, microscopy was used to image cell migration to the scratch.

### Fibrin bead assay (FiBA)

To evaluate the angiogenic potential of Notch peptibodies, 6 × 10^4^ HUVEC cells (Lonza) were used to coat 150 cytodex beads (Sigma) in Endothelial growth media (EGM, Lonza). The endothelial-coated beads were embedded in fibrin gel (3 mg/ml) with either human IgG Fc (Sino Biologics), N1_10-14_Fc, or N4_10-14_Fc. 5 × 10^4^ NHLFB were seeded on top of the fibrin gel in EGM. The media was changed every other day until day 12. The sprout numbers and length were analyzed by Image J (NIH).

### Mice

All mice used in this study were maintained in a pure C57BL/6 J background. Male and female pups were used arbitrarily in these studies.

### Retinal analysis

C57BL/6 mice postnatal day 1 (P1) pups were injected intragastrically with 12.5 mg/kg of recombinant N1_10-14_Fc decoy or Fc for three days (P1-P3). Eyes were isolated at P5 and were fixed in 4% paraformaldehyde (Thermo Fisher Scientific) for 1 h at 4 °C on a nutator. Following fixation, eyes were washed with 1 × PBS solution. Retinas were dissected and permeabilized in 1 × PBS containing 1% BSA (Fisher Bioreagents) and 0.5% Triton X-100 (Fisher Bioreagents) overnight at 4 °C on a nutator. Samples were then immunostained in PBLEC (5% Triton X-100, 1 M MgCl_2_, 1 M CaCl_2_, and 1 M MnCl_2_ in 1 × PBS) overnight at 4 °C with Biotinylated IB4 (1:50; Vector Laboratories, B-1205) and anti-α-SMA-FITC (1:200; MilliporeSigma, F3777). IB4 was detected with streptavidin-conjugated Alexa Fluor 647 (Invitrogen). Immunostained retinas were postfixed with 4% formaldehyde and mounted in Vectashield (Vector Laboratories). Whole-mount retina images were acquired using Leica Dmi8 Platform. All images were analyzed using ImageJ (NIH).

### Statistics

For qPCR analysis, the ΔΔCt method was used to calculate the relative expression using following steps: (1) Normalization to reference gene: ΔCt_GOI_ = Ct_GOI_–Ct_BA_ and (2) Relative expression between conditions: ΔΔCt_GOI_ = ΔCt_EXP_ − ΔCt_CNT_. Unless noted otherwise, t-tests analysis was performed on all quantified data to determine significant differences between groups using GraphPad Prism 9. *P* values less than 0.05 were considered statistically significant. *p* values < 0.05 are shown with one star (*), *p* values < 0.01 with two stars (**), and *p* values < 0.001 with three stars (***). Unless otherwise noted, error bars represent standard error of mean (SEM). All experiments shown were repeated a minimum of three times.

## Supplementary Information

Below is the link to the electronic supplementary material.

Supplementary file1 (PDF 22110 kb)
