# Supplementary material for: Notch1 and Notch4 core binding domain peptibodies exhibit distinct ligand-binding and anti-angiogenic properties
Source: Angiogenesis. 2022 Nov 15;26(2):249–63. doi: 10.1007/s10456-022-09861-6 (PMC10119233; doi:10.1007/s10456-022-09861-6)
Supplement: Supplementary file 1 — Supplementary file1 (PDF 22110 kb) [file 10456_2022_9861_MOESM1_ESM.pdf]

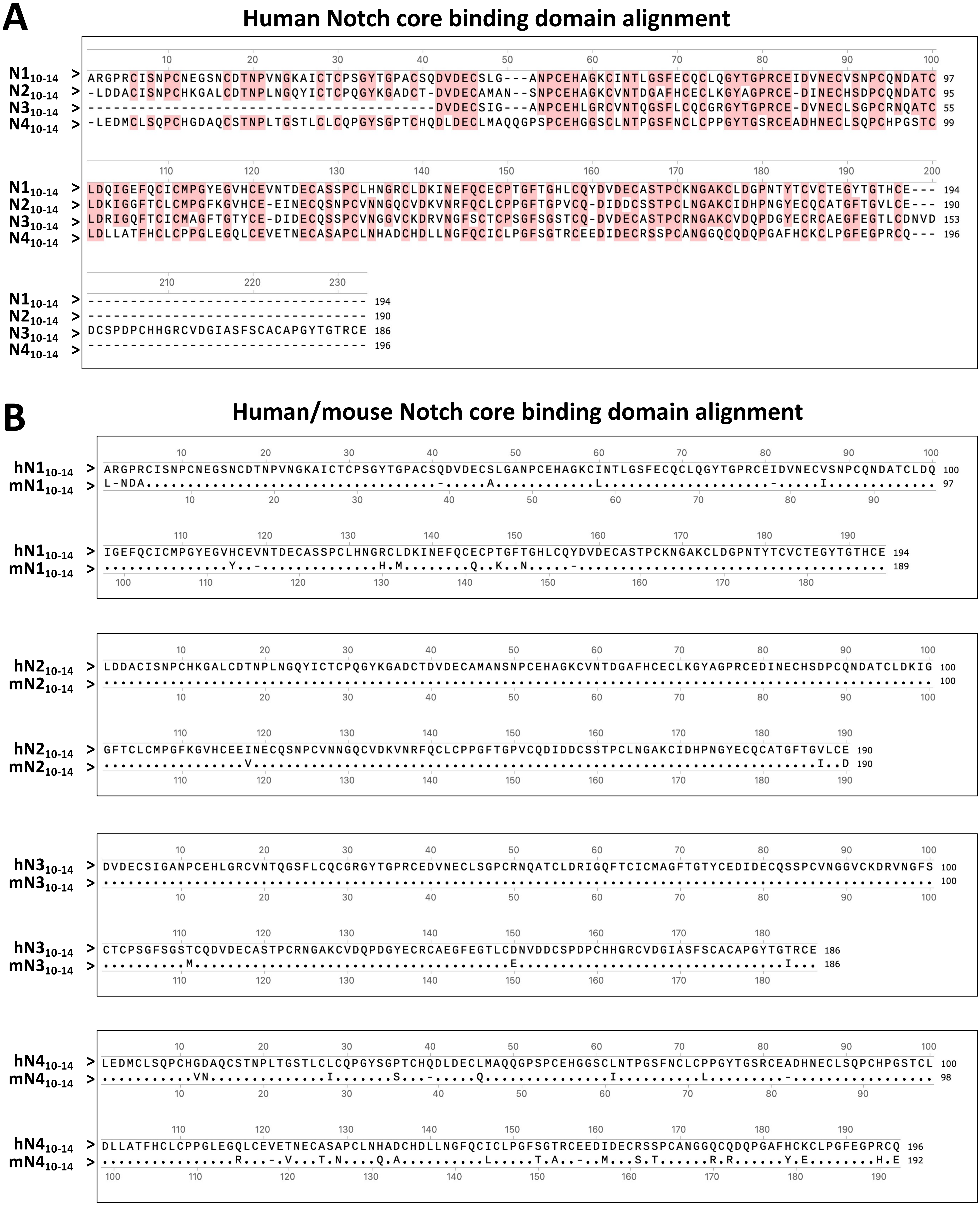

Figure S1: Comparison of Notch putative core binding domains within and between species. (A) Amino acid alignment of EGFs 10-14 of the four human Notch receptors. Pink highlighting indicates identity, dashes indicate lack of alignment. (B) Amino acid alignment of EGFs 10-14 of each human Notch protein with its murine ortholog. Dots indicate amino acid identity.

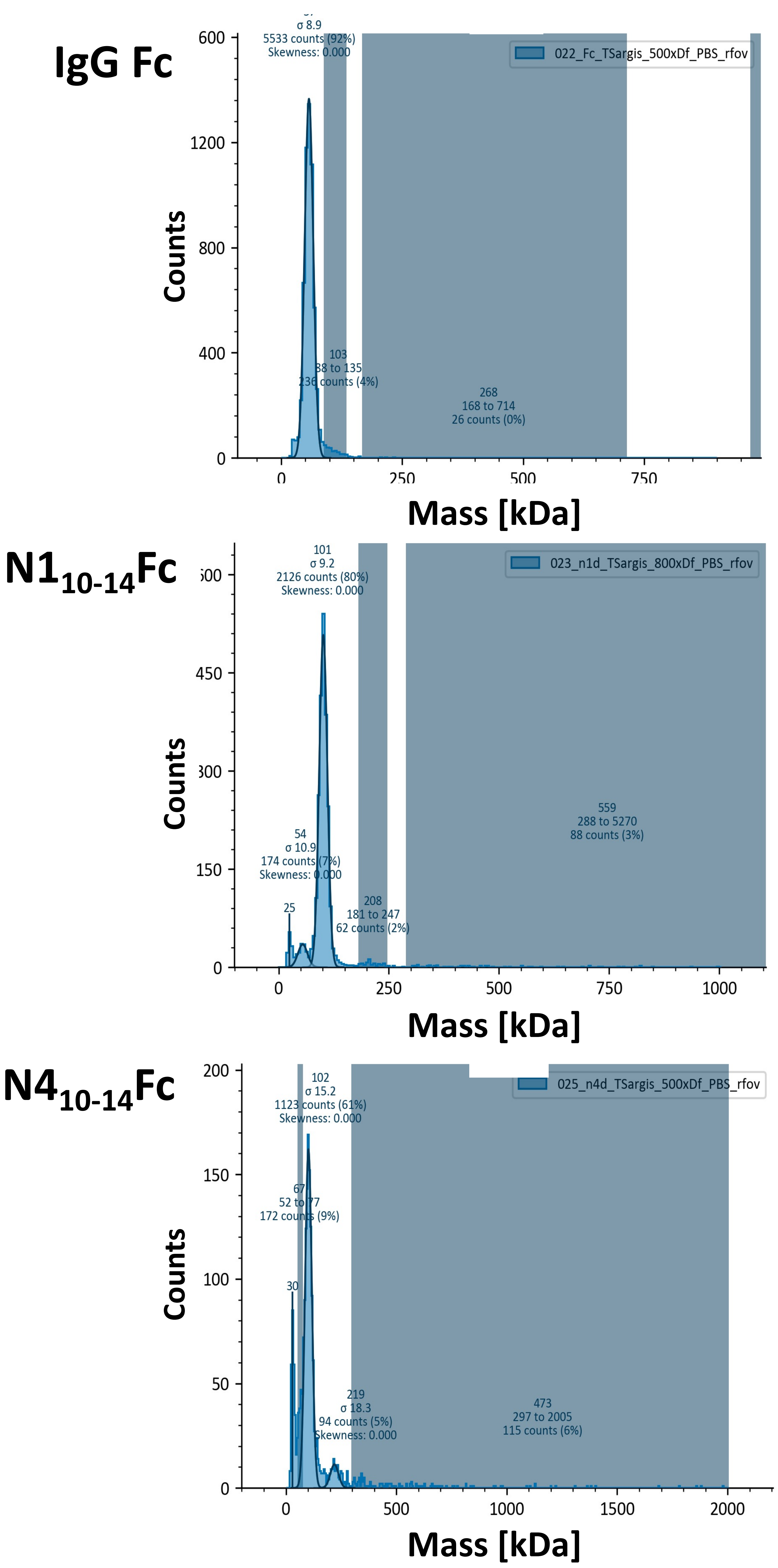

Figure S2: Mass photometry. molecular mass distribution of purified IgG Fc, $N1_{10\text{-}14}$Fc, and $N4_{10\text{-}14}$Fc as measured by mass photometry. Blue shaded regions indicate detectable oligomerization states and their respective percentage of population.

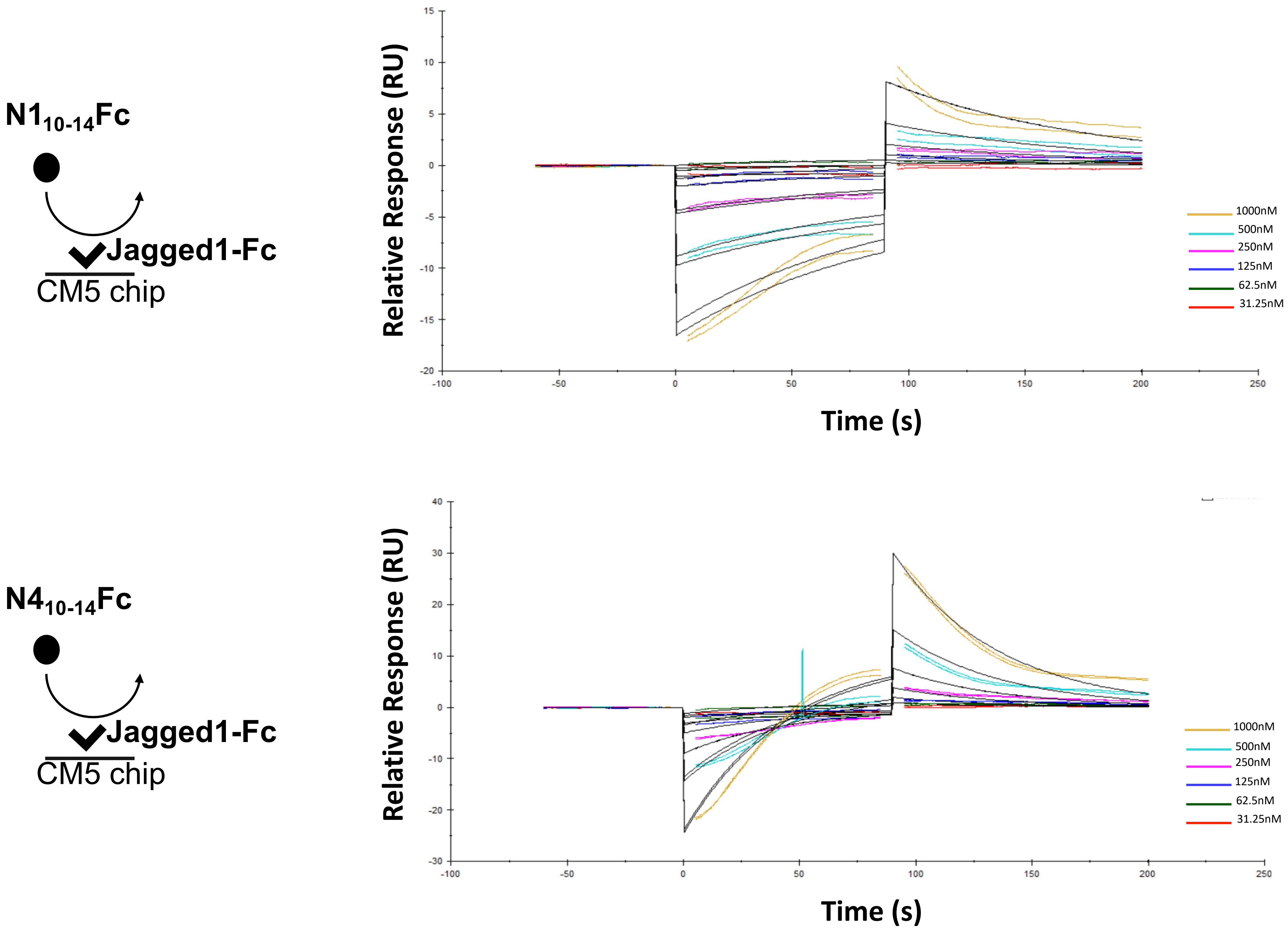

Figure S3: Binding of hJAG1-Fc to $N1_{10\text{–}14}$Fc and $N4_{10\text{–}14}$Fc. SPR measurements show binding affinity of $N1_{10\text{-}14}$Fc and $N4_{10\text{-}14}$Fc to hJAG1. Recombinant Fc-tagged hJAG1 was immobilized on the sensor chip using amine coupling and multi-cycle kinetic experiments were performed using increasing concentrations of either $N1_{10\text{-}14}$Fc or $N4_{10\text{-}14}$Fc. Binding was insufficient to allow for reliable calculation of Kd values.

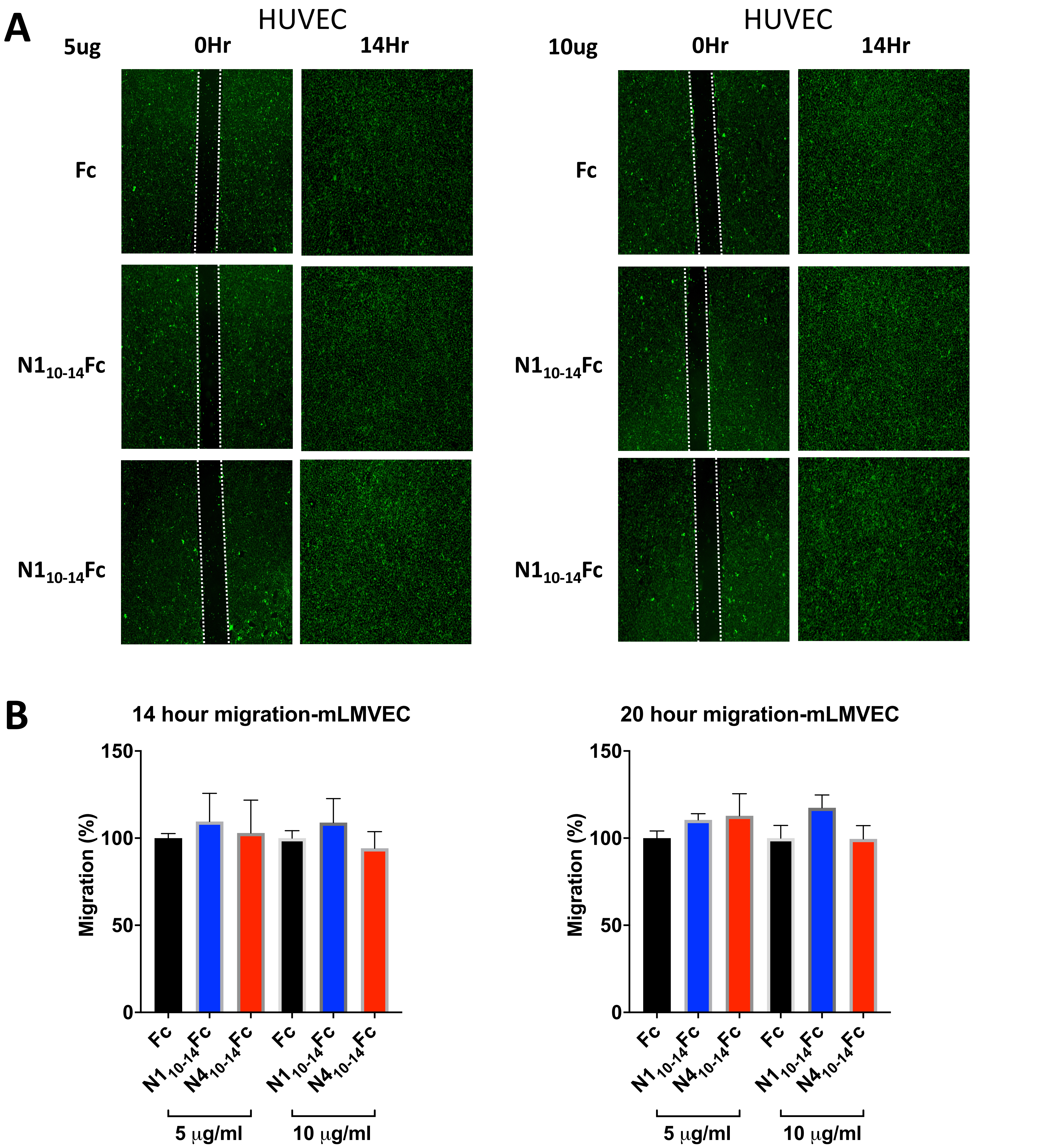

Figure S4: Wound healing assay of endothelial cells treated with Notch peptibodies. (A) HUVEC treated with cell-tracker CMFDA dye were seeded in 24-well plates and "scratch-wounded" using a 200 µl pipette tip. After wounding, cells were treated with different concentrations (5 or 10 ug/ul) of either IgG Fc, $N1_{10-14}Fc$ or $N4_{10-14}Fc$. After 14 hours, microscopy was used to image cell migration into the scratch region. (B) Quantitation of identical experiments performed with mouse endothelial cells (mLMVEC) and assessed at 14 and 20 hours.

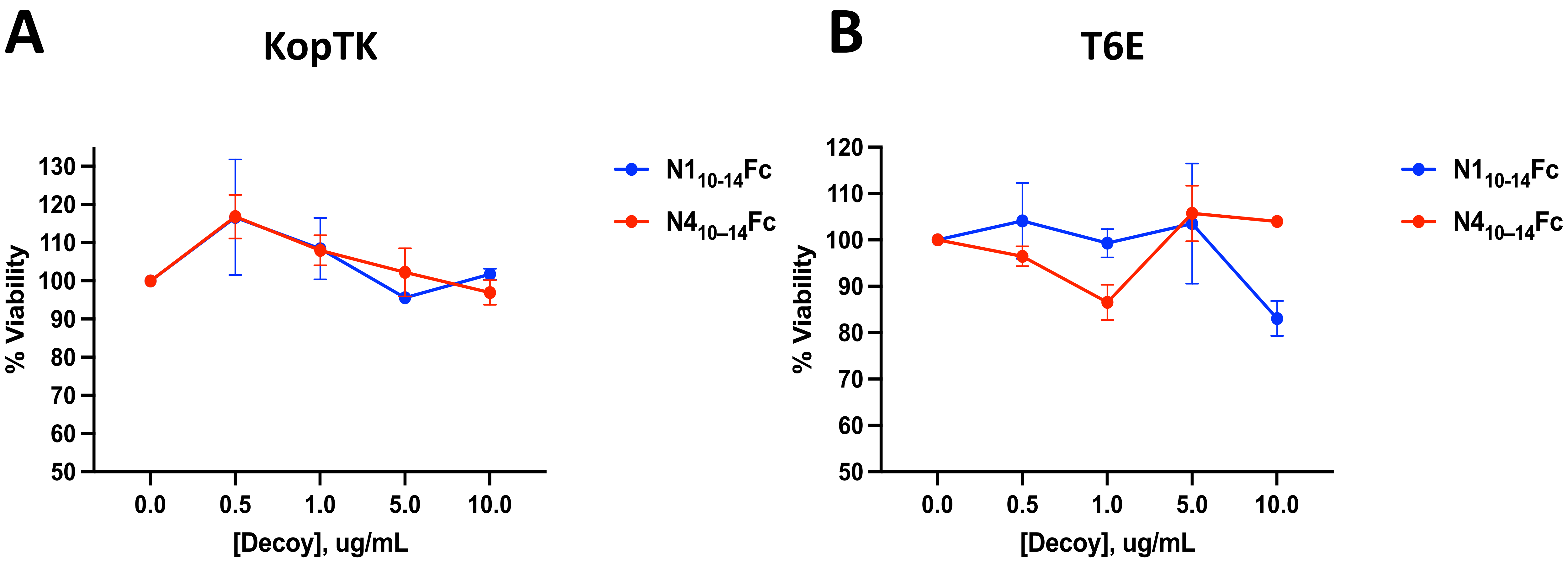

Figure S5: Viability assay of human and mouse T cell acute lymphoblastic leukemia (T-ALL) treated with Notch peptibodies. (A) human KopTK and (B) mouse T6E T-ALL cells were seeded at 8x10^3 cells per well in a 96-well plate in RPMI media and treated with the indicated concentration of IgG Fc, N1$_{10–14}$Fc or N4$_{10–14}$Fc for 72 hours. Viability in peptibody treated cells was assessed as the percent viable cells relative to the IgG Fc treated control group. Error bars indicate standard error of mean. For T-ALL experiments, n=3 for panel A and n=2 for panel B, no differences were significant.

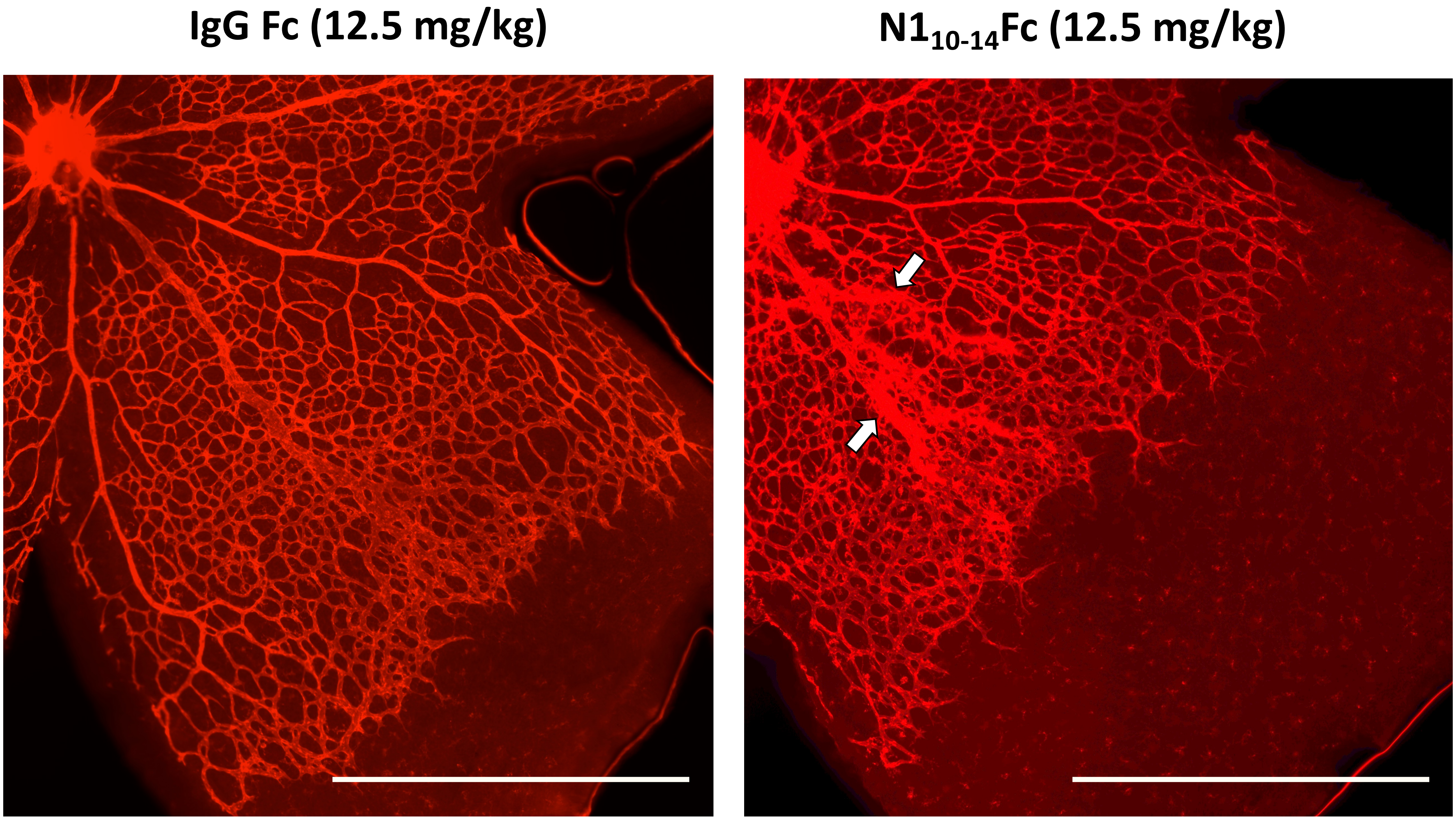

Figure S6: N1$_{10–14}$Fc induces abnormal enlargement of veins *in vivo*. C57BL/6 mice were injected intragastrically with 12.5 mg/kg of recombinant N1$_{10\text{-}14}$Fc decoy or IgG Fc for three days postnatally (P1-P3). (A) Representative images showing post-natal day 5 (P5) retinal vasculature stained with Isolectin B4 (red). (A) Arrowed region highlights enlarged veins. Scale bars: 1000 µm.

TABLE 1: QPCR PRIMERS

Species	Gene	Forward Sequence (5'-3')	Reverse Sequence (5'-3')
**Human**	ACTB	CGAGGCCCAGAGCAAGAGAG	CTCGTAGATGGGCACAGTGTG
**Human**	HEY1	ATCTGCTAAGCTAGAAAAAGCCG	GTGCGCGTCAAAGTAACCT
**Human**	HEY2	GCCCGCCCTTGTCAGTATC	CCAGGGTCGGTAAGGTTTATTG
**Human**	HES1	CCTGTCATCCCCGTCTACAC	CACATGGAGTCCGCCGTAA
**Human**	NRARP	TCAACGTGAACTCGTTCGGG	ACTTCGCCTTGGTGATGAGAT
**Human**	RND1	CTATCCAGAGACCTATGTGCC	CGGACATTATCGTAGTAGGGAG
**Human**	CCL2	AGGTGACTGGGGCATTGAT	GCCTCCAGCATGAAAGTCTC
**Human**	CCL8	TTCTGTGCCTGCTGCTCATG	TTGGATGTTGGTGATTCTTGTGTAG
**Human**	NOTCH1	GTCATCTCCGACTTCATCTACC	CGGAATCAGAGCGTGAGTAG
**Human**	NOTCH4	AGATAAATGGGGGAAAACTGCG	CCTGGGCATCTTTATCGGCT

Table S1: Primers used in RT-qPCR. Table of sequences of qPCR primers.
